# 534. The Use of Tocilizumab and Remdesivir in SARS- CoV-2 Patients in El Paso Texas: A Longitudinal Comparative Observational Study

**DOI:** 10.1093/ofid/ofab466.733

**Published:** 2021-12-04

**Authors:** Nouf K Almaghlouth, Felix Anyiam, Mohamed Attia, Matthew Robinson, Sidra Shah, Syed Haq, Roberto Guevara, Suresh Antony

**Affiliations:** 1 Mountain View Regional Medical Center, Las Cruces, New Mexico; 2 Centre for Health and Development (CHD), Port Harcourt, Rivers, Nigeria; 3 Department of Emergency Medicine, Kasr Alainy University Hospitals, Cairo, Al Qahirah, Egypt; 4 Burrell College of Osteopathic Medicine, Las Cruces, New Mexico; 5 The Hospitals of Providence Transmountain Campus, El Paso, Texas; 6 Texas Tech University Health Sciences Center, El Paso, Texas

## Abstract

**Background:**

Currently, the management of SARS-CoV-2 varies with no definitive clinical guidelines, as scientific evidence across the globe differs in therapeutic options. This study intends to provide some clarity to the insufficient data based on the role of monotherapy with tocilizumab (TCZ) and combination therapy with remdesivir (RDV) and TCZ among patients in El Paso, Texas.

**Methods:**

154 SARS-CoV-2-positive patients from four different hospitals in El Paso, Texas, were screened, with 113 eligible for this longitudinal comparative observational study (2/1/2020-10/31/2020). Group 1 (80 patients) were given TCZ within the first 24 hours of hospitalization, followed by methylprednisolone for 72 hours, and Group 2 (33 patients) received TCZ as detailed in the single therapy group, plus RDV within the first 24 hours. Mann Whitney U test assessed Median differences in laboratory biomarkers and Bivariate Logistic Regression assessed the odds of risk. An observation is said to be statistically significant if P-value is ≤ 0.05.

**Results:**

A statistically significant increased median IL-6 values were noted among those given only TCZ compared to those that received TCZ plus RDV (511.33 vs. 199.0) with *a P-value* (0.007). Patients in Group 1 had statistically significant lower odds for ventilation use than Group 2 (OR=0.34, 95%CI=0.12-0.95, *p=0.034*), although no statistically significant difference in mortality outcomes was observed across groups (OR=0.43, 95%CI:0.13-1.39, p=0.269).

Table 1. Laboratory biomarkers and treatment groups (Mann Whitney U test)

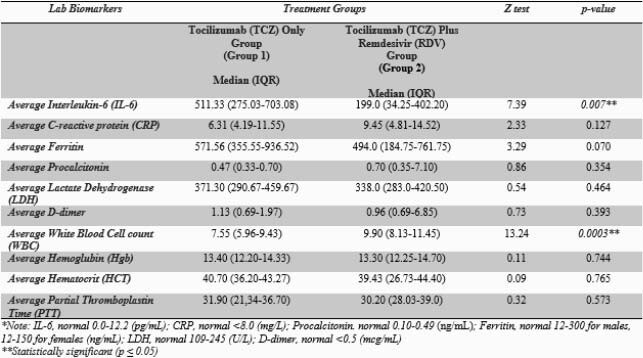

Table 2. Clinical outcomes and treatment groups using the Bivariate Logistic regression (OR)

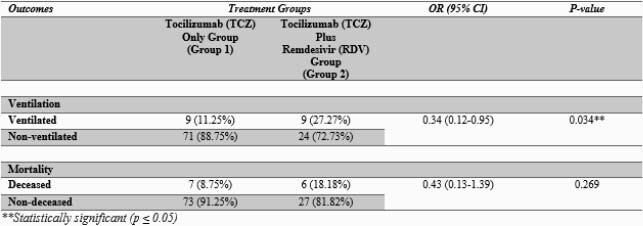

**Conclusion:**

This study population is unique as it reflects a predominantly Hispanic demographic population in El Paso with different genetics, background characteristics, and predisposition to diabetes, and obesity than the rest of the United States (US). We concluded that the use of TCZ in SARS-CoV-2 positive patients in El Paso, with or without RDV, reported no mortality benefit. However, some minimal/non-use of ventilation benefit was observed in Group 1. Our study design is considered the first of its kind using TCZ and RDV in a longitudinal comparative observational study. Nonetheless, a randomized controlled trial study is recommended to ultimately determine the combination role of TCZ and RDV among this highly vulnerable group of patients.

**Disclosures:**

**All Authors**: No reported disclosures

